# Dysphagia as an Initial Presentation of Acute Myeloid Leukemia: A Rare Presentation of Myeloid Sarcoma

**DOI:** 10.7759/cureus.52624

**Published:** 2024-01-20

**Authors:** Rajaa Mohamed Salih, Rahman Olusoji, Chizoba Nwankwo, Noah Osei, Ifeoma P Kwentoh, Obinna T Nwankwo

**Affiliations:** 1 Internal Medicine, Harlem Hospital Center, New York City, USA

**Keywords:** dysphagia, bone marrow biopsy, lymphadenopathy, acute myeloid sarcoma, acute myeloid leukemia (aml)

## Abstract

Myeloid sarcoma (MS) represents a unique clinical presentation of acute myeloid leukemia (AML). This report describes a case of MS in a 66-year-old man who presented with dysphagia, nausea, vomiting, anorexia, and fatigue. Generalized lymphadenopathy was noted on physical exam and confirmed by CT scans which also showed diffuse esophageal wall thickening. Axillary lymph node biopsy was positive for MS. Bone marrow biopsy confirmed AML with 88% blasts. The patient received induction chemotherapy with decitabine and venetoclax and was planned for four cycles of treatment over three months while monitoring the response.

## Introduction

Myeloid sarcoma (MS) historically known as chloromas is a rare lineage of acute myeloid leukemia (AML) known for its green appearance microscopically. MS presents a diagnostic challenge in all populations mimicking almost any systemic disease ranging from gastroenterological (GI) manifestation such as GI bleeding to the least expected urological manifestation even to isolated cardiac myeloid [1]. A high degree of suspicion is necessary in order not to miss these masqueraders. While it is known to involve lymphatics and skin, almost any tissue can be a culprit thus having endless presentations and posing a diagnostic challenge that inadvertently can be missed by clinicians [2]. We present a case of de novo involvement of deglutition. Masking as oropharyngeal dysphagia.

## Case presentation

A 66-year-old male with a history of hypertension, prostate cancer status post robotic surgery, essential thrombocytosis, and chronic kidney disease presented with complaints of generalized weakness, poor appetite, nausea, vomiting, and dysphagia. His dysphagia progressed over one month, initially notable with solids and subsequently with liquids also associated with a choking sensation. The patient had also developed bilateral anterior and posterior cervical and inguinal swellings which he started noticing a few weeks before his presentation and has lost about 8-10 pounds in one month. He reported no recent travel, and no exposure to toxins or pets, but had subjective fevers, and night sweats with chills and rigor.

On physical exam, he was hemodynamically stable, he was found to be pale with prominent superficial and deep cervical lymphadenopathy along the entire cervical chain, he was also found to have bilateral posterior auricular lymphadenopathy and occipital lymphadenopathy. There was no palpable spleen or liver.

**Figure 1:**
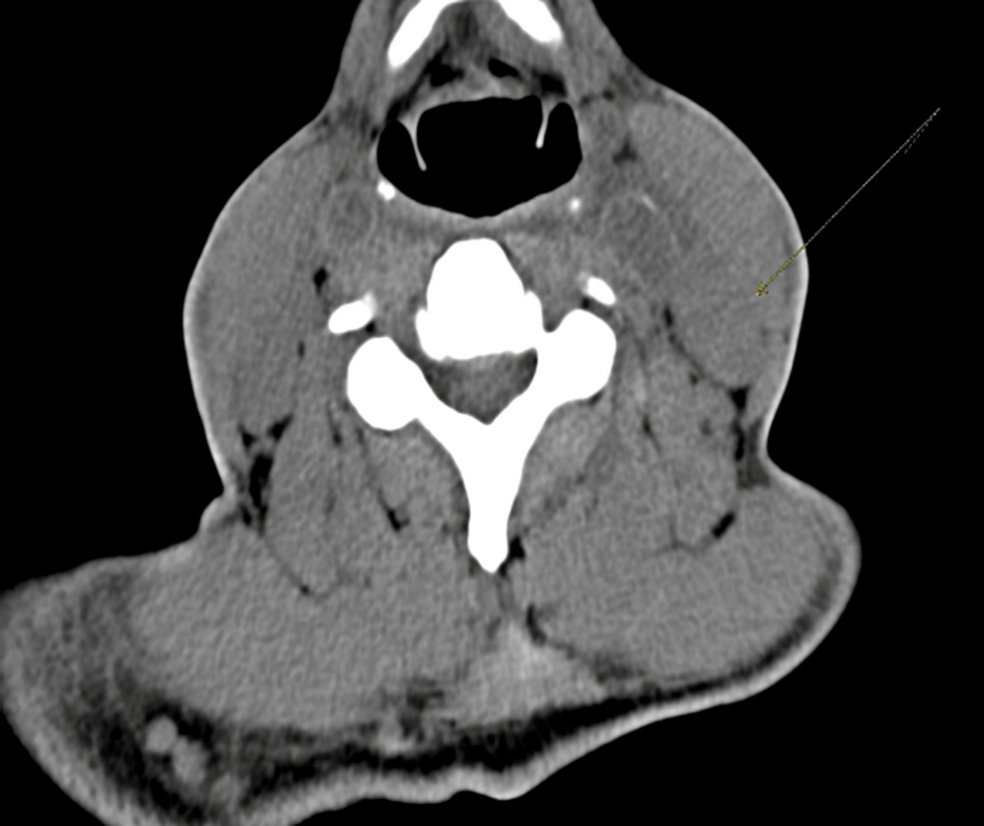
CT scan of soft tissue neck without contrast showing diffuse cervical lymphadenopathy

**Figure 2:**
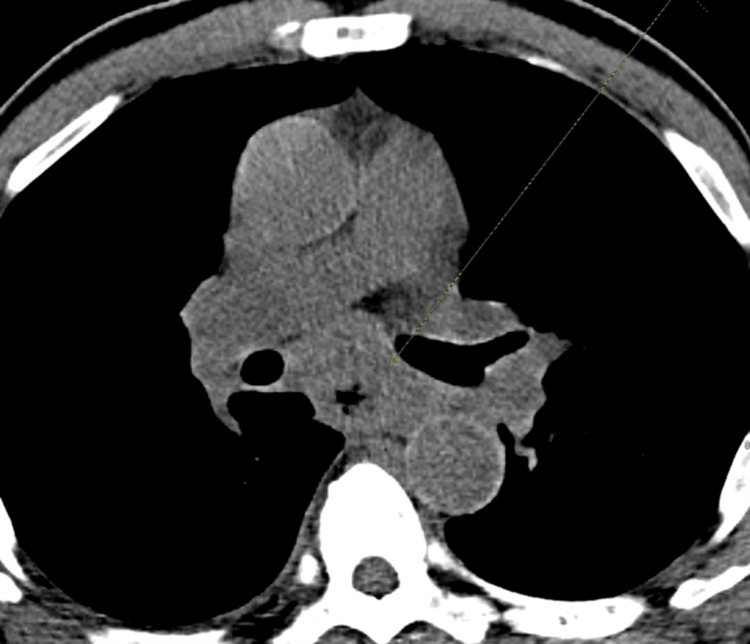
CT chest without contrast showing lymphadenopathy and diffusely thickened esophageal wall

The laboratory results were notable for elevated creatinine of 2.3, and anemia with hemoglobin of 7.6. CT soft tissue neck without contrast showed diffuse cervical lymphadenopathy (Figure 1). CT chest without contrast showed lymphadenopathy, diffusely thickened esophageal wall (differential being inflammatory versus neoplastic infiltration) and calcified left lower lobe granuloma (Figure 2). CT abdomen showed scattered nodular densities in visualized lung bases measuring up to 5 mm, with upper abdominal, retroperitoneal, pelvic, and inguinal adenopathy. It also showed mild wall thickening of distal esophagus, enlarged spleen, and scattered sclerotic foci in osseous structures, the largest measuring 7 mm.

The patient was admitted to the hospital and resuscitated with IV fluids. He was started on a PPI and antiemetics as needed. Workup was initiated to rule out malignancy, and Hematology/Oncology, Gastroenterology, and Interventional Radiology were all consulted. Esophagogastroduodenoscopy showed erythematous mucosa in the cardia gastric mucosal atrophy and normal esophagus. Lymph node biopsy showed MS - immunophenotypic analysis showed a percentage of abnormal cells of 50%-60%, blasts of 50%-60% positive for CD34, CD117, HLA-DR, dim CD13, CD33, CD11c, and CD38. A summary of the immunophenotypic analysis is summarized in Table 1.

**Table 1:** 10-Color flow cytometry analysis for acute leukemia and myeloid/lymphoid neoplasms – sample from right axillary lymph node was consistent with myeloid sarcoma

Section	Details
Interpretation	Right axillary lymph node: Consistent with myeloid sarcoma.
Immunophenotypic Analysis	Abnormal cells (50-60%). Blasts that are positive for CD34, CD117, HLA-DR, CD13, CD33, CD11c, and CD38.
Results - B-Cell Associated	CD19, CD20, CD10, CD22, CD23, CD11c among others with specified percentages.
Results - Myeloid Associated	CD11b, CD13, CD14, CD15, CD16, CD33, and CD64 with specified percentages.

**Figure 3:**
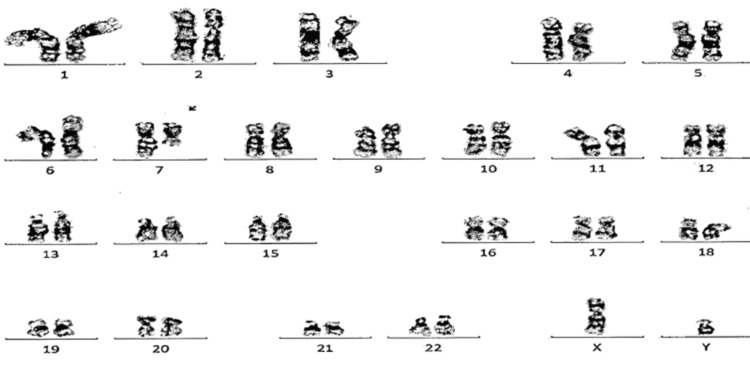
Cytogenetics showing 46, XY, del(7)(q11.2q32)

Bone marrow biopsy showed AML not otherwise specified (AML NOS). Number of blasts (blast equivalents): 88%; phenotype of blasts: CD13+, CD33+, CD34+, CD38+, subset CD117+, subset CD11C+ and HLADR+; marrow cellularity: ~100%; marrow fibrosis: mild patchy (MF-1). FISH was only positive for deletion of 7q (7q-), an abnormality seen in myelodysplasia syndrome and AML (Figure 3) [3].

After the bone marrow biopsy was diagnosed with AML, the diagnosis was shared with the patient, and he opted to start chemotherapy. Intravenous fluids and allopurinol were started in preparation.

He was further evaluated for his kidney function with renal ultrasound showing increased echogenicity. A transthoracic echocardiogram was done before starting chemotherapy and it was unremarkable. He was treated with chemotherapy 3/7 with decitabine for five days and planned for additional chemotherapy.

## Discussion

MS is a tumor mass made of myeloid blasts with or without maturation occurring in an extramedullary site [4]. It may occur in isolation or more commonly in patients with a history of AML, myeloproliferative neoplasm (MPN), myelodysplastic syndrome (MDS), MPN/MDS, or in the blast phase of chronic myeloid leukemia (CML) [5]. MS most commonly occurs in association with AML, affecting 2.5% to 9.1% of patients. It is described as primary or isolated MS when detected before clinical signs of leukemia and associated with a standard bone marrow biopsy and blood film [6]. In our patient, it was the first presentation of AML.

MS symptomatology depends on the tumor’s location, with symptoms usually occurring due to a tumor mass effect or local organ dysfunction [7]. Patients with MS presenting with soft-tissue masses can present a diagnostic challenge given the broad differential diagnoses, including infectious causes and second primary malignancies such as melanoma or Hodgkin's lymphoma [8]. The compression of the esophagus in our patient resulted in dysphagia, a rare presentation of MS, with few reported cases [9,10].

Diagnosis is based on clinical presentation, radiological findings, and tissue biopsy. CT and magnetic resonance imaging (MRI) are often used for tumor localization and help distinguish MS from other entities. Immunohistochemistry is usually used to confirm diagnosis due to clinical and pathological diagnosis challenges [11]. The most commonly expressed antigens are CD34, CD68, lysozyme, Myeloperoxidase (MPO) and CD117, CD11c, CD13 and CD33 [1,5]. Cervical lymph node biopsy and immunophenotypic analysis in our patient showed a percentage of abnormal cells of 50%-60%, blasts 50%-60% positive for CD34, CD117, HLA-DR, dim CD13, CD33, CD11c, and CD38 consistent with commonly expressed antigens for MS [11].

Management depends on factors such as tumor site and size, its relation to local structures, patient age, and performance status [11]. Genetic abnormality should be identified for targeted treatment and determination of prognosis [11]. Systemic chemotherapy is the mainstay treatment even in isolated MS, given that most (71% to 100%) of patients treated with localized methods (surgery and radiotherapy) progress to acute leukemia [12]. AML remission induction chemotherapy regimens have been used in MS, including Idarubicin and Cytarabine; Fludarabine, Cytarabine, Idarubicin and G-CSF (FLAG); Cyclophosphamide, Cytarabine, Topotecan and G-CSF (CAT-G); and Daunorubicin and Cytarabine [7,13]. MS patients treated with AML-type chemotherapy regimens seem to have comparable outcomes to AML patients [7].

## Conclusions

MS is rare and can present in various organs, making diagnosis challenging. It can be considered in cases of atypical tissue infiltration. In our case, dysphagia led to diagnosis, and imaging showed esophageal thickening. MS is associated with AML and requires a multidisciplinary approach to diagnosis. Treatment involves systemic chemotherapy similar to that used for AML and should be commenced early.
